# Characteristics of traumatic brain injury in children and adolescents hospitalized in a Brazilian trauma reference center: a retrospective cross-sectional study

**DOI:** 10.1055/s-0045-1806743

**Published:** 2025-04-22

**Authors:** José Roberto Tude Melo, Caio Vinicius de Almeida Chaves, Cindy Kawano, Maria Antonia Coladeti Fernandes, Reem Hussin, Jean Gonçalves de Oliveira, José Carlos Esteves Veiga

**Affiliations:** 1 Santa Casa de São Paulo, Faculdade de Ciências Médicas, Departamento de Cirurgia, Divisão de Neurocirurgia, São Paulo SP, Brazil.; 2 Santa Casa de São Paulo, Faculdade de Ciências Médicas, São Paulo SP, Brazil.

**Keywords:** Craniocerebral Trauma, Glasgow Coma Scale, Brain Injuries, Wounds and Injuries

## Abstract

**Background** Approximately 20% of victims of traumatic brain injury (TBI) in Brazil are children and adolescents, with more than 900 deaths per year.

**Objective** To describe the characteristics and epidemiological profile of children and adolescents with TBI in the city of São Paulo, Brazil, according to age groups and metropolitan area of occurrence of the events leading to trauma.

**Methods** We conducted a retrospective cross-sectional study with a review of consecutive medical records of children and adolescents with TBI hospitalized and treated at a level-1 trauma center in São Paulo between 2019 and 2023.

**Results** In the period proposed for the study, 196 children and adolescents suffered TBIs. They had a median age of 5 (interquartile range [IQR] 10–1.75) years and were predominantly boys (71%), of white skin color/race (55%), and coming from the north zone of the metropolitan region of São Paulo (44%). Domestic accidents were the main causes of TBI (61%), followed by traffic accidents (24%). The mean length of hospital stay was of 13 (standard deviation [SD] ± 26) days, and the in-hospital mortality rate was of 3%.

**Conclusion** We found a predominance of children and adolescents with TBI coming from the north zone of the metropolitan region of São Paulo, with a prevalence of falls from heights above the ground among children ≤ 9 years of age and trampling among children older than this age. Preventive actions must be established after reflections on socioeconomic issues and considering the metropolitan area where the accidents occur and the age group.

## INTRODUCTION

The incidence of hospitalizations related to traumatic brain injury (TBI) among children and adolescents in Brazil is estimated at 45.35:100 thousand inhabitants/year, with approximately 941 deaths/year. 1 Nearly 20% of TBI victims in Brazil correspond to patients in the pediatric age group. 2 They are victims of home accidents in the case of preschool age children, and traffic accidents in the case of older children, a trend observed worldwide. 1 3 4 5 6 7 Urban sprawl and the consequent motorization of transport has increased the challenges involved in creating public prevention programs in large metropolitan areas. 3 6 7 8 9

The Brazilian Unified Health System (Sistema Único de Saúde, SUS, in Portuguese) is recognized as one of the largest in the world, and it is responsible for the care of approximately 208 million inhabitants. São Paulo is the city with the largest population in Brazil, of approximately 12 million inhabitants, 10 and it has 5 main public hospitals considered neurotrauma reference centers, which are part of the SUS network. 11 The scope of the present study was to identify and describe the cases of children and adolescents with TBI hospitalized and treated at a level-1 trauma center in the central region of the city of São Paulo, with stratification of data by age group, TBI severity, metropolitan area where the accidents occurred, and epidemiological profile of the patients. Our objective is to provide support to targeted preventive actions based on our results, according to the metropolitan area studied.

## METHODS

### Study design, sample, and location

**Figure 1:**
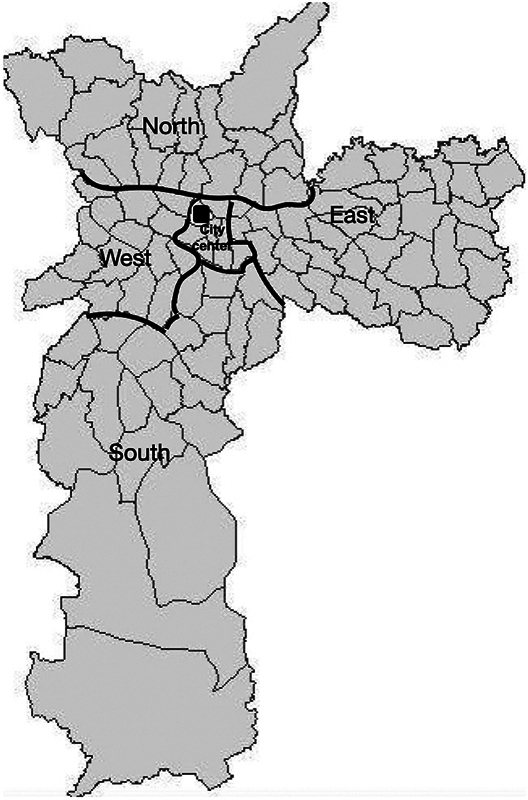
Zoning map of the city of São Paulo 12 with the location of Hospital Central da Santa Casa de Misericórdia de São Paulo (HCSCMSP), a level-1 trauma center.

We conducted a cross-sectional study through the review of consecutive medical records of children and adolescents aged < 17 years with TBI admitted between January 2019 and December 2023 to the Pediatric Emergency Department (PED) of Hospital Central da Santa Casa de Misericórdia de São Paulo (HCSCMSP), a level-1 trauma center 11 located in the Center of São Paulo ( Figure 1 ). The HCSCMSP provides emergency medical care for approximately 2 thousand adult and pediatric patients on a daily basis, covering an area with approximately 500 thousand inhabitants, which increases to an area with approximately 2.78 million inhabitants when neurosurgery care is regarded. 12 13 The TBI patients arrive at trauma centers through an integrated network composed of the Mobile Emergency Care Service (Serviço de Atendimento Médico de Urgência, SAMU, in Portuguese) and the Vacancy Regulation Center (Central de Regulação de Vagas, in Portuguese), which is responsible for the standardization of prehospital care and transportation of patients from the accident site to the reference hospital center. 13 14 15 Victims with severe and moderate TBIs (Glasgow coma scale [GCS] score ≤ 13) treated and transported via VRC-SAMU are intubated and placed on mechanical ventilation when indicated, with cervical immobilization, heated with a thermal blanket, and they have adequate venous access established for fluid infusion and sedation when necessary. 15 16 In cases of mild TBI (GCS scores of 14 and 15), the patients also eventually arrive in the PED by means other than the VRC-SAMU.

Blood samples for laboratory tests are collected immediately upon arrival at the PED in cases of severe TBI and in all cases requiring surgical intervention. In cases of severe TBI (GCS score ≤ 8), a whole-body computed tomography (CT) scan is performed for the investigation of multitrauma, and, in cases of moderate and mild TBIs (GCS score > 8), the investigation is carried out with imaging examinations of the region with suspected injury (plain radiographs and/or CT and/or ultrasound scans). After the brain CT scan, the indication for neurosurgical treatment is determined by the neurosurgeon on duty at the PED. Non-surgical cases with GCS score ≤ 13 and all TBI victims following surgery are managed in a Pediatric Intensive Care Unit (PICU) by a specialized team in the HCSCMSP. In cases of patients with GCS scores ranging from 14 to 15, the pediatric and neurosurgery teams consider the clinical manifestations of the patient and CT scan findings to decide whether hospitalization and eventual admission to the PICU are necessary. Victims of mechanical trauma not associated with TBI or who were discharged within the first 24 hours after trauma, as well as victims hospitalized for other reasons, even if due to trauma, but exempted from reassessment and monitoring by the neurosurgery team, were excluded from the final sample.

### Calibration and completion of the data collection instrument

In order to reduce the risks of selection and observation biases, the research group was calibrated 2 months before the official data collection, filling out the data collection instrument with information from the medical records. The selected medical records were discussed in groups whenever doubts emerged. After resolving the doubts and calibrating the research group, we observed equity in the completion of the data collection instrument and the official data collection was started. The data obtained through the review of medical records were compiled in the the Microsoft Excel (Microsoft Corp., Redmond, WA, United States) and PSPP free software for data analysis (GNU General Public License).

### Data collection instrument

The following variables were included in the data collection instrument:

- General characteristics: age, sex, and skin color/race. The division of age groups was based on previous studies 7 on socioeconomic factors and TBI in the pediatric age group. Regarding the definition of skin color/race, the criteria used were those established by the Instituto Brasileiro de Geografia e Estatística (IBGE, the Brazilian Institute of Geography and Statistics), 17 as described in the medical records reviewed.

- Zone of the metropolitan region of São Paulo where the event leading to TBI occurred: the city of São Paulo is divided into north, south, east, west, and central zones ( Figure 1 ). Each zone has socioeconomic particularities and a set of characteristics that may influence the frequency of accidents. 12 18

- Causes of TBI: traffic accidents were considered to correspond to accidents involving motor vehicles and trampling. Home accidents included falls, collisions against household objects, and domestic violence (including suspected abuse). Urban violence, accidents during sports activities, falls from bicycles, roller skates, skateboards and the like, as well as accidents with unspecified causes, were classified in the category “other causes”.

- Year and season when the accidents occurred.

- TBI severity according to the GCS score: we considered the lowest score in the medical records between the moment of the accident and hospital admission or tracheal intubation. 19 20 We stratified the groups according to severity into mild (GCS score: 14–15), moderate (GCS score: 9–13) and severe (GCS score: 3–8) TBI. 15

- Brain CT scan findings upon hospital admission, considering the information described in the medical record by a senior radiologist and the type of treatment (surgical or non-surgical) established by the neurosurgery team based on this examination.

- Presence of multitrauma according to the results of the imaging tests (plain radiographs, CT scan, and/or ultrasound scan). We considered multitrauma to be present when one or more regions besides the brain (TBI) were affected. 15 21

- Length of hospital stay and in-hospital mortality.

### Statistical analysis

Statistical analyses were performed using PSPP software. Considering that the current is an observational study of prevalence, some results are presented in a descriptive manner, without statistical treatment. The quantitative variables and the continuous variables with normal distribution were expressed as mean ± standard deviation (SD) or and median and interquartile range (IQR) values. The Chi-squared test (with the Yates' correction factor) was used to check the association and significance between pairs of categorical variables, comparing the severe TBI group (GCS ≤ 8) with the mild or moderate TBI group (GCS > 8). The results were considered statistically significant when *p* < 0.05.

### Ethical aspects

The present study was evaluated and approved by the Ethics in Human Research of Irmandade da Santa Casa de Misericórdia de São Paulo (under CAAE 71721423.2.0000.5479 and opinion number 6.341.947). The requirement for individual informed consent forms was waived in view of the fact that the current is an exclusively retrospective study using data from medical records. Considering the retrospective nature of the study, there were no new risks regarding the treatment or management of the selected cases. To avoid data leakage or breach of confidentiality, the names and photos or images that could identify the patients were not exposed.

## RESULTS

**Figure 2:**
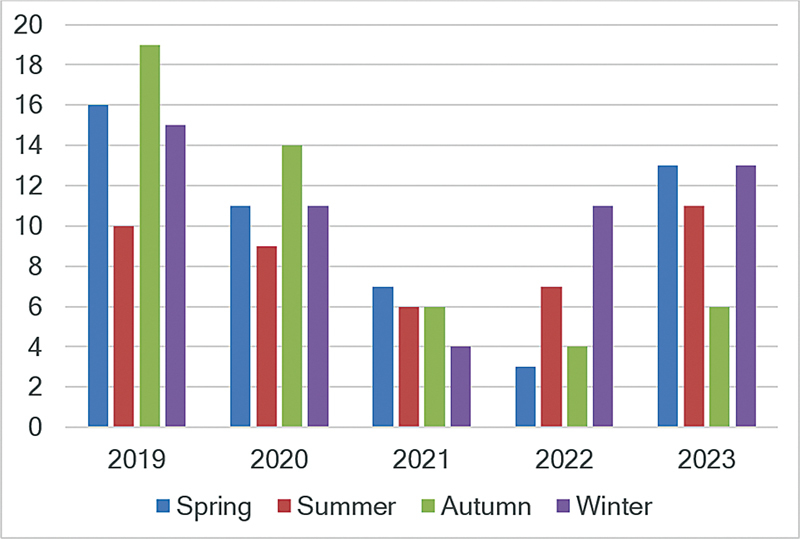
Distribution of the 196 hospitalizations of children and adolescents aged < 17 years with traumatic brain injury (TBI) treated at HCSCMSP from 2019 to 2023.

In the period proposed for the study, 251 admissions via the PED due to TBI were identified, and 196 hospitalizations required monitoring by the neurosurgery team, which was our final sample. The median age was of 5 (IQR: 10–1.75) years, and there was a predominance of male patients (71%; 2.4:1), subjects of white skin color (55%), followed by brown and black skin color (38%), and victims coming mainly from the north zone of the metropolitan region of São Paulo (44%) ( Table 1 ). Domestic accidents (61%; 120/196) predominated over traffic accidents (24%; 48/196) in all zones of the metropolitan region, especially in the east (90%), followed by the central (81%), west (80%), north (70%), and south (66%) zones. Among the domestic accidents, falls from heights above the ground stood out especially in children ≤ 9 years of age (62%; 89/144), while traffic accidents predominated in children older than 9 years (56%; 29/52). We observed a reduction in hospitalizations in 2021 and an increase in the following year, with a heterogeneous distribution across the seasons of the year ( Figure 2 ). Regarding the severity of TBI according to the GCS, there was a predominance of mild cases (68%). Based on this stratification (mild, moderate or severe TBI), we analyzed the main diagnoses found in the imaging exams (brain CT scans) that helped in the decision of treatment to be provided (neurosurgical or not) and in the identification of multitrauma, according to the results of plain radiographs, CT scans and/or ultrasound scans ( Table 2 ). The mean length of hospital stay was of 13 ± 26 days, considering the entire sample, and the in-hospital mortality rate was of 3% ( Table 2 ).

**Table 1:** Profile and epidemiological characteristics of the study sample

General characteristics		Age (in years): n (%)				Total: n (%)
		≤ 4: n = 89 (45)	5–9: n = 55 (28)	10–14 n = 31 (16)	≥ 15 n = 21 (11)	196 (100)
Sex: n (%)	Male	57 (64)	41 (74)	22 (71)	19 (90)	139 (71)
	Female	32 (36)	14 (26)	9 (29)	2 (10)	57 (29)
Skin color/race: n (%)	White	48 (54)	31 (56)	16 (52)	13 (62)	108 (55)
	Black or brown	37 (42)	20 (37)	12 (39)	6 (29)	75 (38)
	Other or not informed	4 (4)	4 (7)	3 (9)	2 (9)	13 (7)
Zone where the accident occurred: n (%)	North	36 (40)	30 (55)	13 (42)	7 (33)	86 (44)
	South	6 (7)	1 (2)	1 (3)	2 (9)	10 (5)
	East	15 (17)	6 (11)	2 (6)	1 (5)	24 (12)
	West	6 (7)	3 (5)	1 (3)	−	10 (5)
	Central	18 (20)	7 (13)	7 (23)	2 (9)	34 (18)
	Unknown	8 (9)	8 (14)	7 (23)	9 (43)	32 (16)
Place of the accident/cause of the trauma: n (%)	Public road	6 (7)	13 (24)	15 (48)	14 (67)	48 (24)
	Car accident	2 (2)	5 (9)	3 (10)	5 (24)	15 (8)
	Motorcycle accident	−	1 (2)	−	3 (14)	4 (2)
	Trampling	4 (4)	7 (13)	12 (39)	6 (29)	29 (15)
	Home accident	73 (82)	37 (67)	8 (26)	2 (10)	120 (61)
	Fall from standing height	4 (4)	5 (9)	1 (3)	−	10 (5)
	Fall from height above the ground ^a^	59 (66)	30 (55)	7 (23)	2 (10)	98 (50)
	Domestic violence	10 (11)	2 (4)	−	−	12 (6)
	Other or undefined causes ^b^	10 (11)	5 (9)	8 (26)	5 (23)	28 (14)
GCS score on admission: n (%)	14–15 (mild TBI)	72 (81)	36 (66)	16 (52)	9 (43)	133 (68)
	9–13 (moderate TBI)	9 (10)	9 (16)	5 (16)	6 (28)	29 (15)
	3–8 (severe TBI)	8 (9)	10 (18)	10 (32)	6 (28)	34 (17)

Abbreviations: GCS, Glasgow Coma Scale; TBI, traumatic brain injury.

Notes: ^a^ Falls from the bed, cradle or similar, bunk bed, chair, caregiver's lap, floor/roof slab, window, trees or walls. ^b^ Other causes: accidents involving skateboars, roller skates, bicycle, scooters, accidents during sports activities, and those due to urban violence (physical aggression).

**Table 2:** Severity of traumatic brain injury according to the Glasgow Coma Scale score and probability of craniocerebral injuries and multitrauma

		GCS score			Total
		14–15 n = 133 (68%)	9–13: n = 29 (15%)	3–8: n = 34 (17%)	n = 196 (100%)
Computed tomography scan: n (%)	Focal brain injury	99 (74)	9 (31)	4 (12)	112 (57)
	Combined ^a^ or diffuse ^b^ brain injury	34 (26)*	20 (69)*	30 (88)*	84 (43)
Proposed treatment for TBI	Non-surgical	112 (84)	23 (79)	21 (62)	156 (80)
	Surgical	21 (16)	6 (21)	13 (38)	40 (20)
Region affected by trauma: n (%)	Isolated TBI	114 (85)	12 (41)	9 (26)	135 (69)
	Multitrauma	19 (15)**	17 (59)**	25 (74)**	61 (31)
Mean length of hospital stay (in days)		6(± 11)	16(± 25)	30(± 47)	13(± 26)
In-hospital mortality: n (%)		−	−	6 (18)	6 (3)

Abbreviations: GCS, Glasgow Coma Scale; TBI, traumatic brain injury.

Notes: ^a^ Brain computed tomography (CT) scan showing combined lesions that determined the treatment established by the neurosurgery team (≥ 2 cranioencephalic injuries described in the results). ^b^ Brain swelling (hypodense brain with reduced or lacking cerebrospinal fluid spaces, but without extravascular hyperdense blood, with a disappearance of the distinction between the white and the gray matters) and diffuse brain contusions (multiple microhemorrhages) were considered diffuse brain injuries. *Combined or diffuse brain injury on CT scan: GCS ≤ 8 versus GCS > 8 (88% versus 33%; Chi-squared = 32.383; 1degree of freedom; *p* < 0.0001). **Diagnosis of multitrauma on imaging exams (plain radiographs, CT scan and/or ultrasound scan): GCS ≤ 8 versus GCS > 8 (74% versus 22%; Chi-squared = 32.158; 1degree of freedom; *p* < 0.0001).

## DISCUSSION

Considering pediatric trauma patients, the median age found in the present study is in agreement with previously published data, bearing in mind that the median age varies depending on the inclusion criteria adopted in each study. For example, the median age varies depending on the range of GCS scores found in the study, on the inclusion or not of infant abuse and cases of firearm projectile injury, among other selection criteria. 1 3 4 5 However, the predominance of boys, especially of school age, is recurrent. 1 3 4 6 Our results are in line with the sex ratio found among pediatric TBI victims in a population-based study conducted in Brazil (2.3:1); 1 however, no conclusive explanation for the predominance of male over female subjects, especially in Brazil, has been presented thus far. 1 10 We did not find studies that provided an explanation for the predominance of male subjects, but researchers often discuss the influence of sexist education, in which boys, unlike girls, are stimulated to engage in “boys' games” and play with “boys' toys”, which involve exposure to risks. 22 23 It is noteworthy that when exposure to traumatic agents is not sex-dependent, no predominance of male subjects is observed. 24

Our results differ from those of the literature data on the predominance of black and mixed-race (non-white) children among TBI victims in Brazil. 1 This divergence may have been influenced by the fact that our sample was composed exclusively of children from the metropolitan region of São Paulo. However, results closer to ours have been observed in a study on adult TBI victims conducted in the same reference center. 25

In the current study, the TBI victims came mainly from the north, central, and the east zones of the metropolitan region of São Paulo (together, they were responsible for 74% of the hospitalizations). The aspects that may have contributed to this result include:

- The coverage area of the studied hospital, which corresponds mainly to the central and north zones; 13

- The fact that the north zone has one of the largest populations of children in the city; and

- The fact that the north and east zones are more affected by poverty and socioeconomic inequalities. 12

Socioeconomic disparities are considered risk factors leading to worse prognosis in children and adolescents with TBI. 7

Falls, one of the main mechanisms of trauma recorded in the present study, stand out in the literature as an important cause of TBI. 3 6 7 8 Regarding falls from great heights, the risk of falls from floor/roof slabs and/or windows, with similar trauma mechanism and severity, was highlighted in two previous works published by one of the authors of the current study (JRTM). 6 8 Slabs are structures used between floors in the construction of buildings as a support for counter-floors or ceilings. In Brazil, concrete floor/roof slabs are often used as areas for socializing, especially in more impoverished urban spaces, often without adequate protection to prevent falls. 26 A change in the main causes of TBI is observed when children reach school age: traffic accidents, especially those involving trampling, become more frequent due to the greater exposure and vulnerability of these children to risk factors outside of their homes. 3 6 16 27

Regarding the frequency of hospitalizations, we observed a reduction in admissions due to domestic and traffic accidents in 2021, with an increase in the following year. This may be related to the period of the coronavirus disease 2019 (COVID-19) pandemic decreed by the World Health Organization (WHO) from March 11, 2020, to May 4, 2023, considering that outdoor mobility restrictions were established during the most critical phase of the pandemic. 28 29 A reduction in pediatric trauma during the pandemic has been reported in other studies, 30 either due to the lockdown imposed or due to the fear of some families to seek medical care to avoid being infected by the severe acute respiratory syndrome coronavirus 2 (SARS-CoV-2). Concerning our results on the heterogeneous distribution of TBI cases across the seasons, the small variations in temperature among the seasons typical of tropical areas likely explain the lack of interference of seasons in hospitalizations in countries like Brazil, 6 and our results do not enable us to make further inferences on this topic.

We found a prevalence of mild TBI, similarly to several previous studies. 3 6 31 Thus, research and the establishment of parameters to guide the interpretation of brain CT scans in mild TBI patients have been more profuse than in other groups. 32 The identification of focal craniocerebral injuries in victims of mild TBI and the greater frequency of combined and diffuse lesions in victims with moderate and severe TBI are important for clinical reasoning and the establishment of actions for the treatment of pediatric TBI patients. In line with our results, the same reasoning can be applied to the investigation of multitrauma, considering the importance of whole-body CT scans in victims of severe TBI. 16 31 32 33

The mean length of hospital stay in the present study was longer than the mean value reported in a previous Brazilian population-based study. 1 The length of hospital stay varies depending on the severity of TBI, the treatment performed, the presence of multitrauma, the need for admission to the PICU, and the care measures established. 34 35 The length of hospital stay found in a sample of adults with mild TBI in the HCSCMSP in a previous study 25 was close to our results when considering only children with mild TBI. We recognize that several variables influence the length of hospital stay among TBI victims, but it was not the scope of the current study to analyze them. Similar to the rate found in the present study, the in-hospital mortality rate found in a Brazilian population-based study 1 including children and adolescents with TBI was of 3.26%. Mortality rates among TBI patients can be influenced by the use of preventive methods at the moment of the accident, pre-hospital care, adequate transportation to the hospital, the reference center considered for analysis, the inclusion of very serious victims who die at the scene of the accident, and the age group considered for these analyses. 1 16 19 20 35 36 37 When only children with severe TBI were analyzed, we also observed consonance with previous studies 16 36 37 conducted in level 1-trauma centers with standardized and routine pre-hospital care, transportation, and hospital care. To the best of our knowledge, no studies in Brazil have analyzed the occurrence of TBI in children and adolescents assisted in level-1 trauma reference centers according to the metropolitan area where the first care measures were provided (accident scene), so as to promote discussions about assertive preventive measures. This is one of the contributions of the present study. We highlight that socioeconomic conditions must be considered when devising trauma-prevention proposals, as emphasized by other authors. 7 The north and east zones together have 70% of the neighborhoods with the worst human development index (HDI ≤ 0.79) among the districts of the city of São Paulo, which has an HDI of 0.84. 38

### Limitations, merits, and perspectives of the study

Considering that the current is a retrospective study with review of consecutive medical records, biases related to data selection, observation, and interpretation were reduced through the pilot study and calibration, which were carried out before the official data collection. We acknowledge that the present study was conducted in a single trauma reference center involving the care and management of children and adolescents with TBI in the city of São Paulo; thus, the results do not necessarily reflect the reality of other reference centers in the state, or even in Brazil. We stress that epidemiological studies must go beyond disclosing percentages of occurrences. The current study envisions the possibility of reaching the public agencies responsible for implementing and regulating preventive methods, seeking to reduce accidents that lead to TBI and consequently the sequelae and deaths resulting from these events. As researchers and physicians, we need to become aware of our collective responsibility towards life, and prevention remains the best alternative when it comes to TBI. Following the publication of the present research, we will seek support from governmental and non-governmental institutions to disseminate our results and raise awareness among the responsible public agencies so that the data herein obtained can be transformed into preventive actions and planning.

In the present study, which was conducted in a single trauma reference center in the city of São Paulo, we conclude that most hospitalized children and adolescents with TBI came from the north zone of the metropolitan region. There was a predominance of domestic accidents, especially falls from heights among children ≤ 9 years, and accidents on public roads, especially those involving trampling, among children above this age. The most severe cases prevailed among victims of trampling accidents, with a higher probability of diffuse brain lesions identified on CT scans and multitrauma. Preventive actions should be developed considering reflections on socioeconomic issues and implemented through strategies aimed at each age group and metropolitan area where accidents occur in order to obtain better results and reduce these traumas.
